# Identification and Characterization of MIKC^c^-Type MADS-Box Genes in the Flower Organs of *Adonis amurensis*

**DOI:** 10.3390/ijms22179362

**Published:** 2021-08-28

**Authors:** Lulu Ren, Hongwei Sun, Shengyue Dai, Shuang Feng, Kun Qiao, Jingang Wang, Shufang Gong, Aimin Zhou

**Affiliations:** College of Horticulture and Landscape Architecture, Northeast Agricultural University, Harbin 150030, China; luluren0808@163.com (L.R.); sunhongwei0720@163.com (H.S.); 15246468669@163.com (S.D.); fengshuang86@163.com (S.F.); kunqiao@neau.edu.cn (K.Q.); wangjingang99@neau.edu.cn (J.W.)

**Keywords:** *Adonis amurensis*, MADS-box genes, ABCDE model, SOC1, flowering, flower organs

## Abstract

*Adonis amurensis* is a perennial herbaceous flower that blooms in early spring in northeast China, where the night temperature can drop to −15 °C. To understand flowering time regulation and floral organogenesis of *A. amurensis*, the MIKC^c^-type MADS (Mcm1/Agamous/ Deficiens/Srf)-box genes were identified and characterized from the transcriptomes of the flower organs. In this study, 43 non-redundant MADS-box genes (38 MIKC^c^, 3 MIKC*, and 2 Mα) were identified. Phylogenetic and conserved motif analysis divided the 38 MIKC^c^-type genes into three major classes: ABCDE model (including AP1/FUL, AP3/PI, AG, STK, and SEPs/AGL6), suppressor of overexpression of constans1 (SOC1), and short vegetative phase (SVP). qPCR analysis showed that the ABCDE model genes were highly expressed mainly in flowers and differentially expressed in the different tissues of flower organs, suggesting that they may be involved in the flower organ identity of *A. amurensis*. Subcellular localization revealed that 17 full-length MADSs were mainly localized in the nucleus: in *Arabidopsis*, the heterologous expression of three full-length SOC1-type genes caused early flowering and altered the expression of endogenous flowering time genes. Our analyses provide an overall insight into MIKC^c^ genes in *A. amurensis* and their potential roles in floral organogenesis and flowering time regulation.

## 1. Introduction

The MADS (Mcm1/Agamous/Deficiens/Srf)-box transcription factor gene family plays an important role in the regulation of plant growth and development [1]. This large gene family is divided into two types, types I and II, based on phylogenetic relationships of the conserved MADS-box domain [2]. In plants, the type-I genes are further divided into Mα, Mβ, and Mγ subfamilies, and the type-II genes into the MIKC^c^-type and MIKC*-type [3,4]. The term MIKC originated from the four major domains, including MADS (M), intervening (I), keratin-like (K), and C-terminal (C) [5].

The MIKC^c^-type MADS-box genes are involved in flowering time regulation and floral organ identity. For example, flowering locus C (FLC), suppressor of overexpression of constans1 (SOC1), and short vegetative phase (SVP) were reported to be key regulators of flowering time; the *FLC* gene encodes a specific MADS domain protein that acts as a repressor of flowering, and SOC1 and SVP are important control factors of flowering time in the vernalization and ambient temperature pathways, respectively [6,7,8]. Moreover, the MADS-box genes of the extended ABCDE model explain how the different floral organ identities belong to the MIKC^c^ subgroups [9], namely, sepals (A + E), petals (A + B + E), stamens (B + C + E), carpels (C + E), and ovules (D + E). In this model, class A contains APETALA1 (*AP1*) and FRUITFULL (*FUL*); class B contains PISTILLATA (*PI*) and APETALA3 (*AP3*); class C contains AGAMOUS (*AG*); class D contains SEEDSTICK (*STK*); and class E contains SEPALLATA genes (*SEP1*, *SEP2*, *SEP3*, and *SEP4*) [9]. The ABCDE model was initially established in *Arabidopsis* and also works for most eudicots.

The identification and characterization of MADS-box genes are extremely important for the study of flowering time regulation and flower organ development in plant species. Currently, MADS-box genes were identified and characterized in various plant species with reference genomic resources, including *Arabidopsis* [5], rice [10], *Zea mays* [11], soybean [12], *Raphanus sativus* [13], *Phyllostachys edulis* [14], and *Jatropha curcas* [15]. However, gene identification in non-model plant species without genomic resources is difficult. Recently, multiple functional MADS-box genes were identified and characterized in *Lilium formosanum* and *Rosa chinensis* using transcriptome sequencing [16,17], suggesting the feasibility of this method. *A. amurensis* is a perennial herbaceous flower in the family Ranunculaceae, which is naturally distributed in northeast China. *A. amurensis* can blossom before the ice and snow melts in the early spring, when the temperature is about −15 °C (night) and 10 °C (day) [18]. Therefore, it is the ideal plant species to study flowering control at extreme low temperatures. In this study, 43 non-redundant MADS-type transcripts were extensively identified from transcriptomes of flower organs at multiple development stages. Further, the conserved motifs, expression patterns, and subcellular localization of the expressed proteins were investigated. Moreover, the function of three SOC1-type *MADS* was characterized by heterogenous expression in *Arabidopsis*. This study will serve as a useful reference for further functional analyses of candidate genes involved in the flowering time control and flower development of *A. amurensis* at low temperatures.

## 2. Results

### 2.1. Identification and Annotation of MADS-Box Genes in A. amurensis

**Figure 1:**
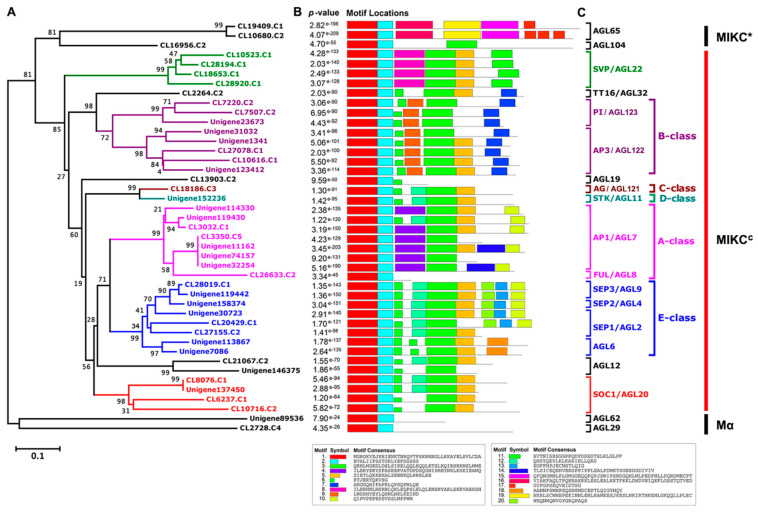
Classification and conserved motifs of MADS-box putative proteins in *Adonis amurensis.* (**A**) The phylogenetic tree of the identified 43 AaMADS putative proteins and their conserved motifs. (**B**) Motifs 1 to 20 are indicated by different colored boxes. The combined probability values are shown on the left side. (**C**) Classification and naming of 43 AaMADS putative proteins based on the similarity to *Arabidopsis* MADS-box homologous proteins. SVP: Short vegetative phase; AG: Agamous; AGL: Agamous-like; TT16: TRANSPARENT TESTA 16; PI: PISTILLATA; AP: APETALA; STK: SEEDSTICK; FUL: FRUITFULL; SEP: SEPALLATA; SOC1: Suppressor of overexpression of CONSTANS1; MIKC: MADS (M), intervening (I), keratin-like (K), and C-terminal (C) domain.

In our previous study, the 3216 transcription factors (TFs) in the *A. amurensis* flower organs at six developmental stages were identified and classified by transcriptome sequencing. Of these TFs, 91 MADS-type transcripts were annotated [18]. After removing the redundants, 43 MADS-box putative genes were finally obtained (Table S1). The phylogenetic tree and conserved motifs of these 43 *AaMADS* putative genes were constructed and identified (Figure 1A,B). Among the corresponding proteins, motifs 1 and 2 were identified and were conserved MADS domains. Motifs 3 and 5, which were keratin (K) domains, were identified in 27 AaMADS proteins (Figure 1B). The 43 AaMADS putative proteins were named and classified according to the phylogenetic relationship between AaMADS and *Arabidopsis* MADS (also known as the Agamous-like, AGL) proteins. They were subdivided into 3 major classes, Mα, MIKC*, and MIKC^c^, of which MIKC^c^ was divided into 7 subclasses, including SVP (four members), A-class (AP1 and FUL, eight members), B-class (PI and AP3, eight members), C-class (AG, one member), D-class (STK, one member), E-class (SEP1/2/3 and AGL6, eight members), and SOC1 (four members) (Figure 1C). Interestingly, only two MIKC*-type AtAGL65 homologous proteins (CL19409.C1 and CL10680.C2) shared motifs 15, 16, 17, and 19 (Figure 1C).

### 2.2. Expression of AaMADS Genes in the Flowers of A. amurensis

**Figure 2:**
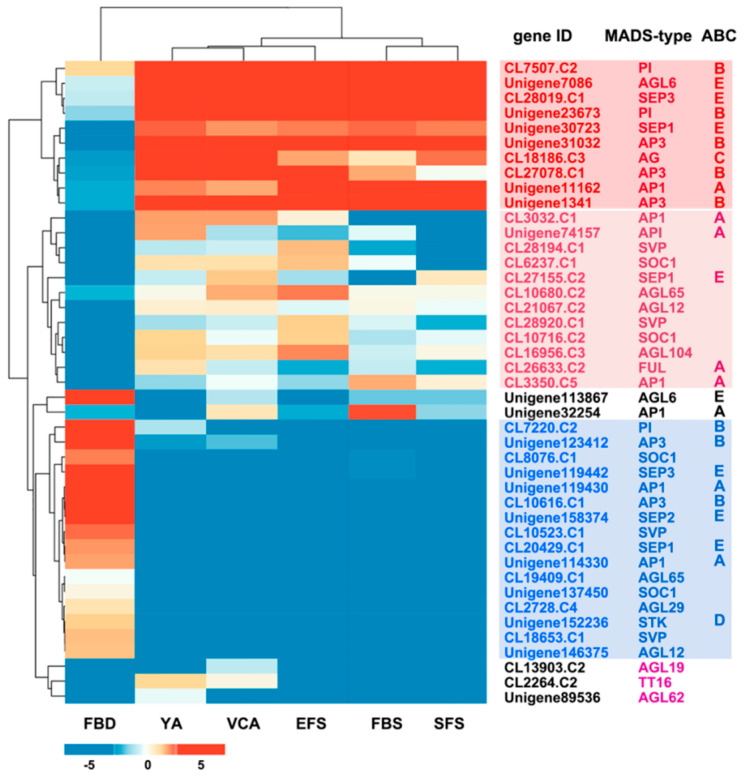
Expression pattern clustering of 43 *AaMADS* putative genes in floral organs at six developmental stages (FBD: flower bud differentiation; YA: young alabastrum; VCA: visible color alabastrum; EFS: early flowering stage; FBS: full bloom stage; SFS: senescing flower stage). The levels of expression of each gene during FBD, YA, VCA, EFS, FBS, and SFS are indicated by red/blue rectangles, where red rectangles represent the upregulation of genes, while blue rectangles represent downregulation.

**Figure 3:**
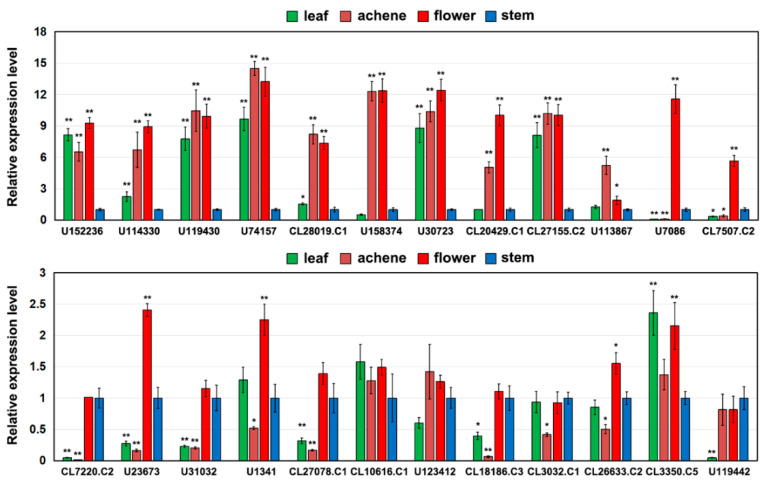
Expression analysis of 24 *AaMADS* genes in different tissues, including stems, leaves, flowers, and achene by qPCR. The *AaActin* gene was used as an internal control, and the transcript level in stems was set as 1.0. Asterisks indicate significant differences in gene expression levels between other tissues and the stem (* *p* < 0.05; ** *p* < 0.01; Student’s *t*-test). Error bars represent the *SE* (*n* = 3).

**Figure 4:**
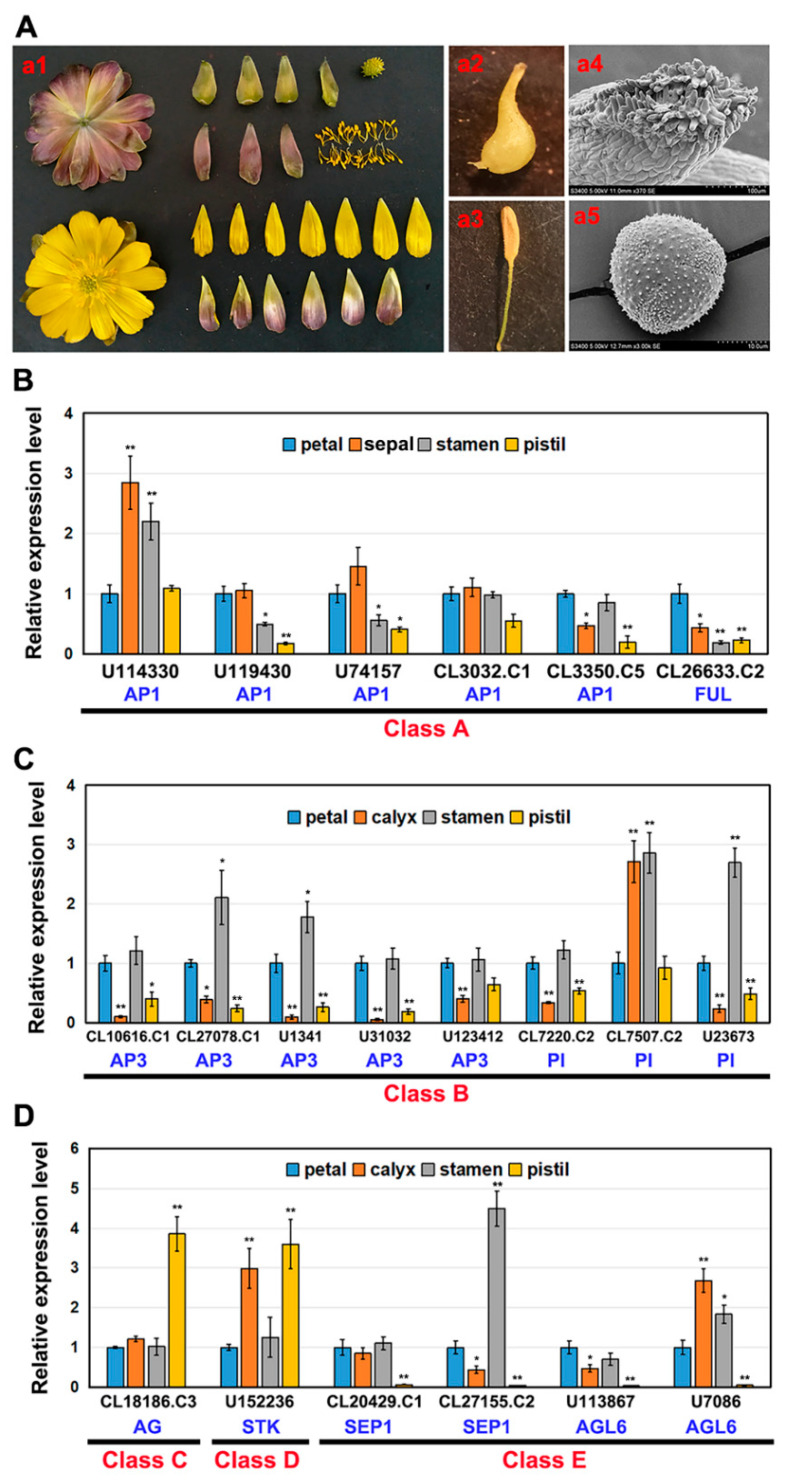
Organ-specific expression analysis of 20 *AaMADS* genes classified in the ABCDE model in the flower of *A. amurensis*. (**A**) The flower morphology and its anatomy, including petals, calyx, stamens, and pistils (**a1**), simple pistil (**a2**) and stigma (**a4**), simple anther (**a3**) and pollen grain (**a5**). qPCR analysis of A-class (**B**), B-class (**C**), and C-, D-, and E-class (**D**) *AaMADS* gene expression in different flower structures. The *AaActin* gene was used as an internal control, and the transcript level in petals was set as 1.0. Asterisks indicate significant differences in gene expression levels between other tissues and petals (* *p* < 0.05; ** *p* < 0.01; Student’s *t*-test). Error bars represent the *SE* (*n* = 3).

Based on transcriptome data, the expression of the 43 *AaMADS* genes showed two different patterns in the *A. amurensis* flower organs at six developmental stages. One is the high expression of *AaMADS* genes in five development stages [young alabastrum (YA), visible color alabastrum (VCA), full bloom stage (FBS), and senescing flower stage (SFS)], while the other is the high expression of *AaMADS* genes only in the flower bud differentiation (FBD) stage (Figure 2). To understand their expression patterns, the expression of 24 *AaMADS* genes classified in the ABCDE model in stems, leaves, flowers, and achenes was examined. qPCR analysis showed that *AaMADS* genes belonging to the A-, B-, C-, D-, and E-classes were generally higher expressed in flowers than in stems, leaves, and achenes (Figure 3). Further, their expression in the four tissues of the flower organs was investigated. *A. amurensis* flower includes about 7–9 sepals (pale grayish purple), about 10–13 petals (yellow), ellipsoid ovary, stigma unsmooth, sac-like anther, and spherical pollen grains (Figure 4A). In the class A genes, five AP1 (*U114330*, *U119430*, *U74157*, *CL3032.C1*, and *CL3350.C5*) were expressed slightly higher in the petals, sepals, and stamens, while one FUL (*CL26633.C2*) was expressed higher in the petals (Figure 4B). In the class B genes, five AP3 (*CL10616.C1*, *CL27078.C1*, *U1341*, *U31032*, and *U123412*) and two PI (*CL7220.C2* and *U23673*) were highly expressed in the petals and stamens, while one PI (*CL7507.C2*) was highly expressed in the sepals and stamens (Figure 4C). One C-class gene AG (*CL18186.C3*) and one D-class gene STK (*U152236*) were highly expressed in the stamens (Figure 4D). In the class E genes, one SEP1 (*CL27155.C2*) was highly expressed in the stamens, while one SEP1 (*CL20429.C1*) and two AGL6 (*U113876* and *U7086*) were highly expressed in the petals, sepals, and stamens (Figure 4D).

### 2.3. Subcellular Localization of AaMADS Proteins

**Figure 5:**
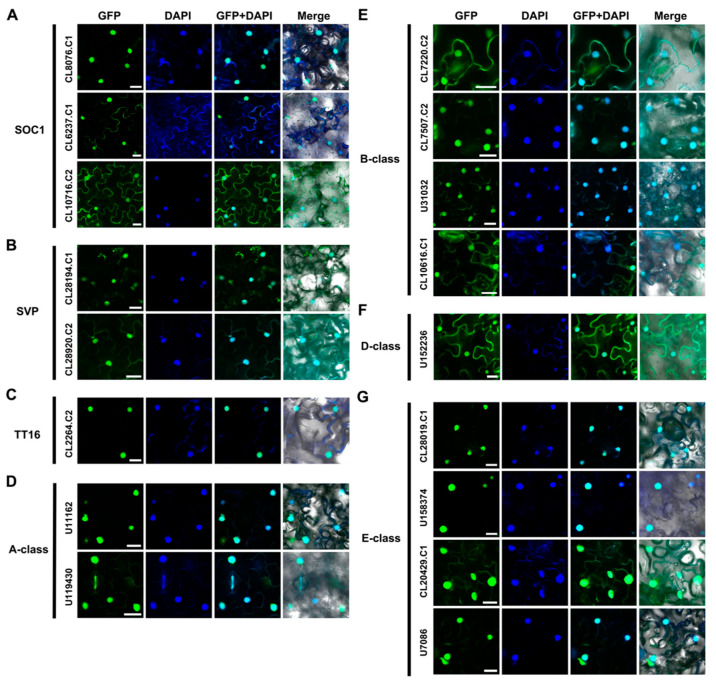
Subcellular localization of 17 AaMADS fused with green fluorescent protein (GFP) in tobacco leaves. The 17 AaMADS belonged to the SOC1 type (**A**), SVP type (**B**), TT16 type (**C**), A-class (**D**), B-class (**E**), D-class (**F**), and E-class (**G**) groups, respectively. GFP fluorescence is green and the nuclear dye DAPI is blue. Merge is created by merging the GFP, DAPI, and bright-field images. Scale bar = 10 µm.

The subcellular localization of 17 full-length AaMADS proteins classified in the SOC1-, SVP-, TT16-type, and ABCDE model was investigated by transient expression with green fluorescent protein (GFP) fused with AaMADS proteins in tobacco leaves. Confocal observations showed the fluorescent signals of all 17 AaMADS-GFP, including three SOC1 (CL8076.C1, CL6237.C1, and CL10716.C2), two SVP (CL28194.C1 and CL28920.C2), one TT16 (CL2264.C2), two A-class (U11162 and U119430), four B-class (CL7220.C2, CL7507.C2, U31032, and CL10616.C1), one D-class (U152236), and four E-class (CL28019.C1, U158374, CL20429.C1, and U7086), were mainly localized in the nucleus, which was stained by DAPI (Figure 5A–G).

### 2.4. Characterization of Three SOC1-Type AaMADS Genes

**Figure 6:**
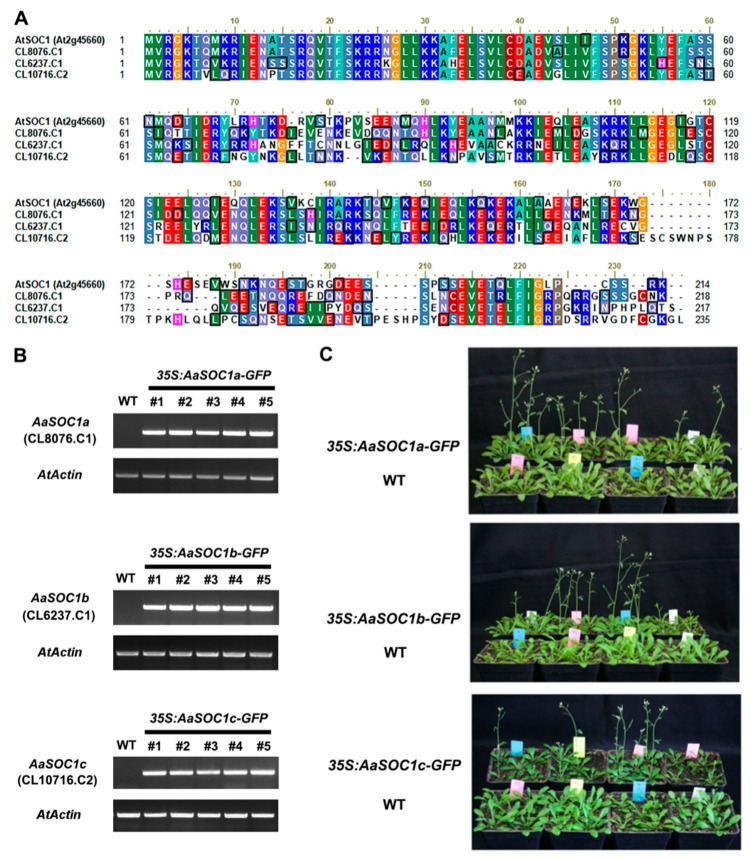
Phenotypic analysis of transgenic *Arabidopsis* overexpressing three SOC1-type *AaMADS* genes. (**A**) Amino acid sequence alignment of three SOC1-type AaMADS (CL8076.C1, CL6237.C1, and CL10716.C2) with *Arabidopsis* AtSOC1 (At2g45660) protein. Semi-quantitative PCR detection (**B**) and flowering time phenotypes (**C**) of transgenic lines overexpressing three SOC1-type *AaMADS* genes. The *AaActin* gene was used as an internal control. WT: Wild-type.

**Figure 7:**
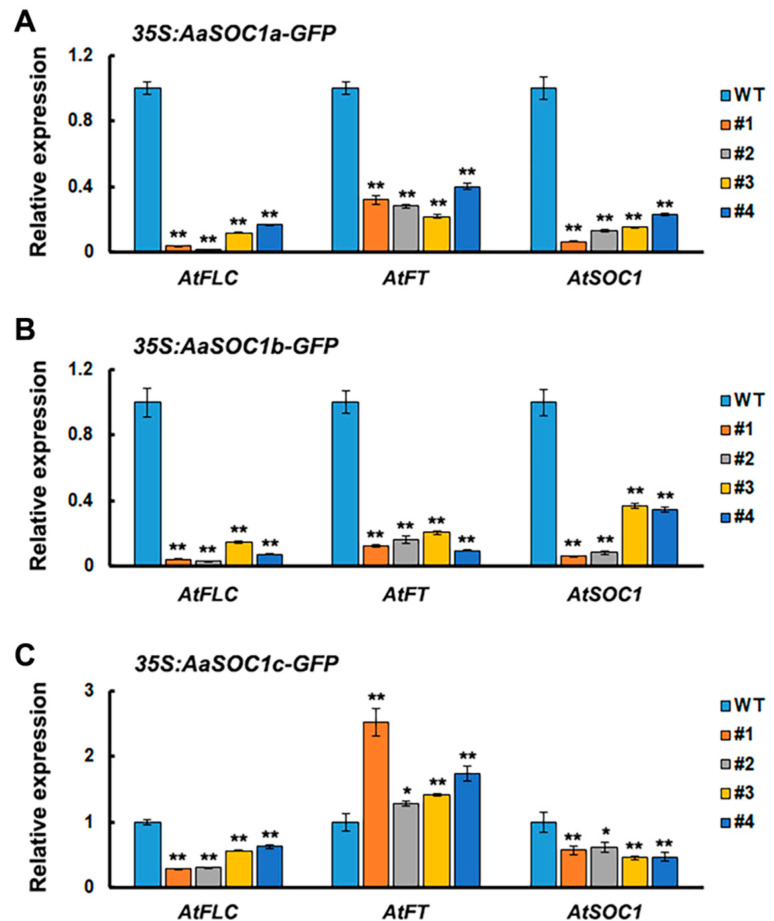
Relative expression level of endogenous flowering time genes in wild-type (WT) and transgenic *Arabidopsis* overexpressing *AaSOC1a*- (**A**), *AaSOC1b*- (**B**), and *AaSOC1c-GFP* (**C**) genes. The *AtActin* gene was used as an internal control, and the transcript level in WT was set as 1.0. Error bars represent the SE (*n* = 3). Asterisks indicate significant differences between transgenic lines and WT plants (* *p* < 0.05; ** *p* < 0.01; Student’s *t*-test). FLC: Flowering locus C; FT: Flowering locus T.

SOC1 is a key flowering regulator, which was reported to be associated with the final steps of floral organ development [19]. Thus, the function of three full-length SOC1-type *AaMADS* (*CL8076.C1*, *CL6237.C1*, and *CL10716.C2*) was investigated by heterologous expression in *Arabidopsis* driven by the CaMV 35S promoter. CL8076.C1, CL6237.C1, and CL10716.C2 had 66.4%, 53.1%, and 56.3% amino acid sequence identity with AtSOC1, respectively (Figure 6A), and were thus named AaSOC1a, AaSOC1b, and AaSOC1c, respectively. The transient expression of the plasmid of GFP-fused AaSOC1a/b/c showed that they were localized into the nucleus in tobacco leaves (Figure 5A). These constructs were further transfected into *Arabidopsis*. Transgenic *Arabidopsis* lines overexpressing AaSOC1a-GFP, AaSOC1b-GFP, and AaSOC1c-GFP were identified by reverse transcription (RT) PCR (Figure 6B and Figure S1). Phenotypic observations showed that transgenic plants (30.5 ± 0.7 days) overexpressing AaSOC1a-GFP, AaSOC1b-GFP, and AaSOC1c-GFP flowered earlier than the wild-type (WT) control (40.3 ± 1.5 days) (Figure 6C). Furthermore, the expression of endogenous flowering time genes, *AtFLC*, *AtFT*, and *AtSOC1*, in transgenic *Arabidopsis* and WT was compared by qPCR, and results showed that expression of *AtFLC* and *AtSOC1* was significantly lower in all transgenic *Arabidopsis* than in the WT (Figure 7A–C). The *AtFT* expression was likewise significantly lower in transgenic *Arabidopsis* overexpressing AaSOC1a-GFP and AaSOC1b-GFP than in the WT, while it was higher than the WT in transgenic *Arabidopsis* overexpressing AaSOC1c-GFP (Figure 7). These results suggest that the overexpression of AaSOC1a/b/c perturbates the expression of endogenous flowering time genes in *Arabidopsis*.

## 3. Discussion

**Figure 8:**
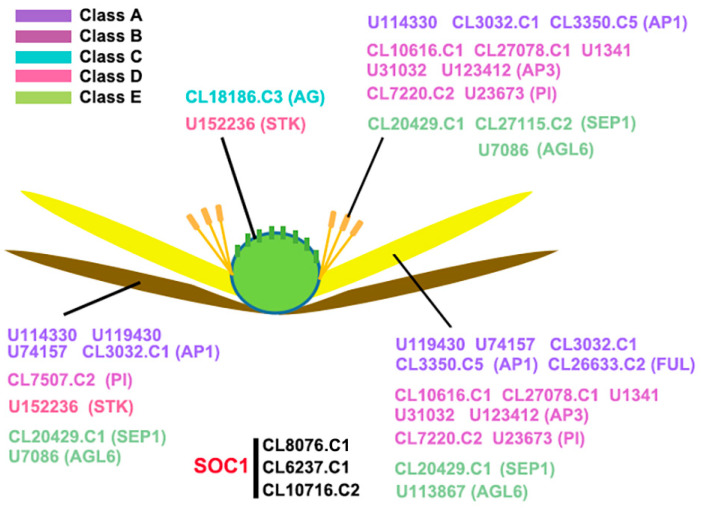
Hypothetical model of expression pattern of *AaMADS* genes. Five colors represent five class *AaMADS* genes, including class A, B, C, D, and E.

The number of the MADS-box genes identified in various plant species shows great difference. For example, in *Arabidopsis* and rice, 107 and 75 MADS-box genes were annotated, respectively, while 42 and 39 MADS-box genes were identified in *Phyllostachys edulis* and *Dianthus caryophyllus*, respectively, using a genome-wide search [5,10,14,19]. Using transcriptional sequencing, 58 MADS-box genes were identified in the flower buds of *R. chinensis* [17]. Similarly, in our study, 43 MADS-box genes were identified from the transcriptional data of *A. amurensis* flower organs using transcriptional sequencing. Both *A. amurensis* and *Aquilegia coerulea* belong to the family Ranunculaceae. In *A. coerulea* from Ranunculaceae, 47 MADS-box genes were annotated using a genome-wide search [20]. The MIKC-type members are the most common in the MADS-box gene family. Among the 107 MADS-box genes in *Arabidopsis*, 39 are MIKC-type [5], whereas among the 75 genes in rice, 38 are MIKC-type [10]. Meanwhile, the identified 43 *AaMADS* genes in *A. amurensis* contain 38 MIKC^c^-type and 3 MIKC*-type (Figure 1). The vast majority of the identified AaMADS are MIKC^c^-type members, which may be related with identifying genes from the flower organs of *A. amurensis*, as MIKC^c^ is primarily involved in flowering time regulation and flower organ identity [6,7,8,9]. The 38 MIKC^c^ genes contain 26 ABCDE model genes and 4 SOC1-type genes. qPCR analysis showed that 24 *AaMADS* genes belonging to the ABCDE model were expressed relatively high in the flowers (Figure 3), while in the petals, the expression of A- (*AP1* and *FUL*), B- (*AP3* and *PI*), and E- (*SEP1* and *AGL6*) class genes was slightly higher. Class A (*AP1*) and E (*AGL6*) genes were expressed slightly higher in the sepals. In the stamens, class B (*AP3* and *PI*) and class E (*SEP1*) genes showed high expression, while the class C (*AG*) and D (*STK*) genes were highly expressed in the pistils (Figure 4). Gene expression of A-, B-, C-, D-, and E-class in *A. amurensis* showed similarities and differences found in *Arabidopsis* and other species. These results suggest that the ABCDE model genes may be involved in the flower organ identity of *A. amurensis*. Thus, a model of gene expression patterns in *A. amurensis* is proposed (Figure 8).

Some AaMADS sequences lack a full-length CDS due to the limitations of transcriptome sequencing. We successfully cloned 17 full-length *AaMADS* and observed their subcellular expression localization. Transient expression in the tobacco leaves showed that the 17 AaMADS-GFP were all localized mainly in the nucleus, suggesting their function as TFs (Figure 5). The 17 full-length *AaMADS* genes contain 3 SOC1-type *AaMADS*, named *AaSOC1a*, *AaSOC1b*, and *AaSOC1c*. SOC1 is a key transcription factor that regulates flowering time [21]. Heterologous expression of *AaSOC1a-GFP*, *AaSOC1b-GFP*, and *AaSOC1c-GFP* all caused the early flowering of *Arabidopsis* (Figure 6). Similarly, heterologous expression of *SOC1* homologous genes from various plant species promotes the early flowering of *Arabidopsis*, for example, *Z. mays* [22], *P. violascens* [23], and *Dendrobium nobile* [24]. In transgenic *Arabidopsis* overexpressing *AaSOC1a-GFP*, *AaSOC1b-GFP*, or *AaSOC1c-GFP*, the *AtFLC* gene expression was significantly suppressed compared to the WT (Figure 7). *AtFLC* expression inhibition is a key step in the flowering of *Arabidopsis* [6]. The expression of *AtSOC1* in all transgenic *Arabidopsis* was also significantly lower than the wild type. Moreover, the expression of flowering locus T (*AtFT*) was also affected relative to the WT (Figure 7). We speculated that *AaSOC1* may functionally replace endogenous flowering time genes, such as *AtSOC1* and *AtFT*, that further affect the flowering time of *Arabidopsis*. However, this hypothesis requires further study.

## 4. Materials and Methods

### 4.1. Identification of MADS-Box Genes in A. amurensis

In our previous study, the transcriptome of the floral organs of *A. amurensis* from six developmental stages, FBD, YA, VCA, EFS, FBS, and SFS, was assembled using Trinity [18]. All assembled unigenes were annotated by comparing the data available at the following public databases: NCBI non-redundant protein sequence (Nr), NCBI nucleotide sequence (Nt), Swiss-Prot protein, Kyoto Encyclopedia of Genes and Genomes (KEGG), euKaryotic Ortholog Groups (KOG), InterPro, and Gene Ontology (GO) databases, using BLAST2GO analysis with a cut-off *E*-value of 10^−^^5^. TFs in the annotated unigenes were predicted using the Plant Transcription Factor Database (PlantTFDB; available online: http://planttfdb.gao-lab.org/. TFs with the same annotated information and the longest unigene were selected. Finally, sequences of 43 MADS-box putative genes were obtained from the transcriptional datasets of *A. amurensis* floral organs. The transcriptome datasets were deposited in the NCBI Gene Expression Omnibus with accession number GSE126456.

### 4.2. Conserved Motifs and Phylogenetic Analysis of MADS-Box Proteins

The conserved motifs of the 43 AaMADS putative proteins were identified by the multiple expectation for motif elicitation (MEME) tool (available online: https://meme-suite.org/meme/) according to the default parameters. Multiple sequence alignments were performed between MADS-box protein sequences from *A. amurensis* and *Arabidopsis* using the ClustalW software. The sequence of the *Arabidopsis* MADS protein family was taken from the TAIR website (available online: https://www.arabidopsis.org/). The phylogenetic tree was constructed by the neighbor-joining method using the molecular evolutionary genetics analysis (MEGA) 4.1 software (available online: http://www.megasoftware.net/). The amino acid sequences of the 43 putative AaMADS proteins are listed in Supplementary Data 1.

### 4.3. Expression-Pattern Clustering of AaMADS Genes

The expression levels of 43 *AaMADS* putative genes in *A. amurensis* floral organs at six developmental stages (FBD, YA, VCA, EFS, FBS, and SFS) were calculated using the Fragments Per Kilobase of transcript per million mapped reads (FPKM) (Table S2).

### 4.4. Quantitative Real-Time PCR (qPCR) Analysis

Total RNA from multiple organs (stems, leaves, flowers, and achene) and tissues (calyx, petals, stamens, and pistils) of *A. amurensis* was extracted using the TRIzol reagent (9108, TaKaRa, Kusatsu, Japan) and reverse transcribed with PrimeScript RT reagent Kit with gDNA Eraser (RR047A, TaKaRa, Kusatsu, Japan). The expression of the 24 AaMADS genes was investigated by qPCR. The primers for these assays (Table S3) were designed using Primer 5.0, and qPCR was performed using a CFX96 real-time PCR detection system (Bio-Rad, Hercules, CA, USA) and SYBR Green PCR Master Mix (RR420A, TaKaRa, Kusatsu, Japan) according to the manufacturer’s instructions. *AaActin* was used as a reference gene. Three biological and three technical replicates were performed for each sample.

### 4.5. Gene Cloning, Vector Construction, and Plant Transformation

Gene cloning was performed by PCR using specific primers (Table S4). To construct the AaMADS-GFP fusion genes, the open reading frame of 17 *AaMADS* genes without the stop codon was amplified using PCR and cloned at the XbaI/BamHI and AgeI/KpnI sites of the pBI121-GFP vector using specific primers (Table S5). The above constructs were confirmed by sequencing and transformed into *Agrobacterium tumefaciens* strain EHA105 for plant transformation. Transient expression in tobacco (*Nicotiana benthamiana*) leaves was performed as previously described [25]. *Arabidopsis* (Columbia ecotype) plants were transformed using the floral dip method [26]. Transgenic *Arabidopsis* plants were selected on 1/2 strength Murashige and Skoog (MS) medium containing 30 μg mL^−1^ kanamycin. Expression of *AaMADS* genes in the transgenic *Arabidopsis* was assessed by semi-quantitative RT-PCR analyses. The T3 generation was used for the phenotypic analyses. *Arabidopsis* seeds were treated at 4 °C for 2 days and then grown on 1/2 MS medium under long-day conditions (16 h light/8 h dark) at 22 °C for 10 days before being transplanted into soil. The light intensity of the growth chambers was 150 µE m^−^^2^s^−^^1^.

### 4.6. Subcellular Localization

The tobacco leaf epidermis was visualized using confocal laser scanning microscopy (CLSM; Nikon, A1, Tokyo, Japan). The nucleus was labeled using a DAPI (4′,6-diamidino-2-phenylindole) dye. GFP and DAPI signals were detected under 500–530 and 420–480 nm emission filters, respectively.

## 5. Conclusions

*A. amurensis* is a perennial plant that flowers under natural conditions at extremely low temperatures, which makes it a potential model for investigating the effects of temperature on flowering regulation in other angiosperms. Among the TFs involved in flowering regulation, the MADS-box genes stand out for their central role. Based on transcriptomic and phylogenetic analyses, we found 43 novel MIKC^c^-type MADS-box genes involved in the regulation of organogenesis and floral development in *A. amurensis*, including genes involved in the ABCDE model, SVP and SOC1. Moreover, heterologous expression in *Arabidopsis* of three of these SOC1-like genes was shown to induce early flowering, suggesting their critical role in the regulation of flowering time in *A. amurensis*. Further, loss-of-function, overexpression, and ectopic expression studies are needed to elucidate the processes regulated by each of these MADS-box genes, as well as the particular features of flowering regulation in *A. amurensis*.

## Data Availability

All the raw sequencing reads for all the samples have been submitted to the National Centre for Biotechnology Information (NCBI) Gene Expression Omnibus with accession number GSE126456.

## Supplementary Materials

The following are available online at https://www.mdpi.com/article/10.3390/ijms22179362/s1.
