# Supplementary material for: Identification and Characterization of MIKCc-Type MADS-Box Genes in the Flower Organs of Adonis amurensis
Source: Int J Mol Sci. 2021 Aug 28;22(17):9362. doi: 10.3390/ijms22179362 (PMC8430553; doi:10.3390/ijms22179362)
Supplement: Supplementary file 1 [file ijms-22-09362-s001.zip › Supplementary data 1.pdf]

>Unigene113867

MGRGRVELKRIENKINRQVTFSKRRNGLLKKAYELSILCDAEVGLIIFSSRGKLYEFASA

GMNRTLERYQRCCYTPQDTTAADRETQSWYQEVSKLKAKYESLQRSQRHLLGEDLGPLSV

KELQNLEKQLEGALAQARQRKTQIMMEQMEELRRKERQLGDINKQLKNKLETEGQGTFRA

IQSSWESTALVASNNFSVHPSQSNSMDCEPTLQIGYHQYVQQEGTNVPRSVGGGGSENNF

MQGWVL

>Unigene23673

MGRGKIEIKRIENLTNRQVTYSKRRNGILKKAKEITVLCEAQVSLVIFSSTGKMAEFSSS

PMITILDKYQTSSGNKLWDAKHEYLSSEVERVKRENDNMQIQLRHLKGEDITSLNPKELI

PIEDALLNGLTKVRDKKTEFCNRMKKNGRMLEEENKRLKYMLLTPEQKMEMEEKADLENG

YHQNSRNYPSQIPFSLCVQPIHPNLQELKDHSY

>CL8076.Contig1

MVRGKTQMKRIENATSRQVTFSKRRNGLLKKAFELSVLCDADVALIVFSPRGKLYEFSSS

SIQTTIERYQKYTKDIEVENKEVDQQNTQHLKYEAANLAKKIEMLDGSKRKLMGEGLESC

SIDDLQQVENQLERSLSHIRARKSQLFREKIEQLKEKEKALLEENKMLTEKNGPRQLEET

NQQREFDQNDENSLNCEVETRLFIGRPQRRGSSSGCNK

>CL8076.Contig2

MVRGKTQMKRIENATSRQVTFSKRRNGLLKKAFELSVLCDADVALIVFSPRGKLYEFSSS

SIQTTIERYQKYTKDIEVENKEVDQQNTQHLKYEAANLAKKIEMLDGSKRKLMGEGLESC

SIDDLQQVENQLERSLSHIRARKSQLFREKIEQLKEKEKALLEENKMLTEKNGPRQLEET

NQQREFDQNDENSLNCEVETRLFIGRPQRRGNVQILS

>CL7220.Contig2

MGRGKIEIKRIENSTNRQVTYSKRRNGIIKKAKEITVLCDAKVSLVIFSSTGKMSEYCSS

TNTGESRLIEILDRYQKSSGKKLWDAKHEYLSAEVDRIKKENDSMQIELRHLKGEDITSL

QPKELIPIEKALQDGFDSVKAKVSEIWKMHKRNLRLAEEEQNRLIFKCQQQQIEIDEVVR

DQMAVNGTGGGGYDHKAAAGTDYPPSHHHQRQMPFAFRGQPLQPNLHNHNK

>CL10680.Contig2

MGRVKLKIKRLENTSGRQVTYSKRRAGILKKAKELSILCDIDIALLMFSPTGKPTLCLGE

RSTIEEVIAKFAQLTPQERAKRKLESLEALKKTFKKLDHDVNIQEFLGSSTQTVEDLTSQ

SRLLEGQLSEMHKRLSCWTEPEKINNLEHLRAMEESLRESLHRIRTHKENLGKQQLLPLD

CTGQFQNAMHLPLGMGGEQQVQPISWIPSSDGQHLMLPEDPNLLHQRDMECPTEASVPGY

SGYFGPPKQVEINPSGQNGGYFDPSKQVEIDTSGQNGSGGGYFGPTKQLEIDTCGHELNR

NACLRLQLGGQYPYPQQYGLNFLLDNKNMKSPEVKMNIQQSQSQSQSQSPLDYQVVANVG

FEPPRLGGYDINRPTWGPNTVVPCANGLFDDHMYPQQHH

>CL10616.Contig1ll

MGRGKIEIKRIENATNRQVTFSKRRSGIFKKARELSVLCDAEVSLIIVSSTNKVSDYISP

STTAKKIFDKYQEVEKIDLWASHYEELQNNLNHQKEINSKLRKEIRQRKGEDLNELSIKE

LRGLEQNLATSLETVRIRKFHVLNTQTDTCKKKIRSLEDAYKTNLRIAEERDEESHYVGP

DNEDDDHYASSSITRLSNYGGGGSYLSFHLKPRQPSLQNAAGEGYSYNGIPLLE

>CL6237.Contig2

MVRGKTQVKRIENSSSRQVTFSKRRKGLLKKAHELSVLCDADVSLIVFSPSGKLHEFSNS

SMQKSIERYRRHANGFFTCNNLGIEDNLRQLKHEVAACKRRNEILEASKQRLLGEGLSTC

SREELYRLENQLERSISNIRQRKNQLFTEEIDRLKEQERTLIQEQANLRECVGQVQESVE

QREIVPYDQSSENCEVETELFIGRPGKRINPHPLQTS

>CL16956.Contig2

MGRVKLQIKRIENNTNRQVTFSKRRNGLIKKAYELSVLCDIDIALIMFSPSGRLSHFSGK

RRIEDVLTRYINLPDHDRGGSIQNREYLIRTLKKLKTESDMAIQIANPTAVNTHIEQLQQ

EITSFQQQIQMTGEQLRNFEPDLLKLTSMNELDACEKQLVEVLTRVSQKKSYLLSNHLST

YDPSSLQMYLDSQEGMVPSNENKDVNWFPENGNNPNQIFVGHNALVPLRDQQLGMYDPLS

QGTSLNIDPHSIEAFQISNQNDQAVPPCHQVYTSTELLNALVPPGPFLLFQPEMEGPEIL

WMIPPEQAETLPNGTIVSTQNNDDVTYQSNSQQQIDVE

>Unigene31032

MGRGKIEIKKIENPTNRQVTYSKRRAGILKKAKELTVLCDAEVSLIMFSSTGKLHEFVSP

STTTKNIYDRYQHAAGVSLWQSHYERMQENLNKLKEINRKLRREISQRNGEELDGLNFQQ

LCGLEQNMEKSAECARNRKFHVIGTQTDTYKKKLLLQVKNHEETQNDLLREFQRRDAECQ

YAAFVDQEGEYQSTVGMANGNVNGHIFAFRLQPSQPNLQHDEGYGSYGLTLA

>CL6237.Contig1

MVRGKTQVKRIENSSSRQVTFSKRRKGLLKKAHELSVLCDADVSLIVFSPSGKLHEFSNS

SMQKSIERYRRHANGFFTCNNLGIEDNLRQLKHEVAACKRRNEILEASKQRLLGEGLSTC

SREELYRLENQLERSISNIRQRKNQLFTEEIDRLKEQERTLIQEQANLRECVGQVQESVE

QREIVPYDQSSENCEVETELFIGRPGKRINPHPLQTS

>CL20429.Contig1

MGRGKVEMKRIENNINRQVTFAKRRNGLLKKAYELSVLCDAEIALIIFNSRGKLYEFCSS

SSILKTLEKYQRSTSSPSQSTTPPKNPEDETYQDYLKLKSRYEVLQQNQRNLNGLDLEPL

SVKELDQLENQLEGSLREIRSTKTQFMMDHLTELKTKQEKELKEVNKAMNRKQLEESKKM

MIPQLKWEPEEHKVFYNHHGSASVDATSEGFYHPIQCNSTMHIGNSSSMRTNVSTGPTPQ

QSAIEYFPGWMI

>CL7507.Contig2

MGRGKIEIKRIENSTNRQVTYSKRKNGILKKAKEITVLCDAMVSLIIFSGSGKMVEYASR

PLGEILDNYHKIAGRKLWDAKHEYLHSEVERVKKENDSMQIELRHLKGEDLTSLNPKELI

PIEEALTNGLAKVREKKNEFVKVMKKNGRMLEEENKRLAYIMEKQQMEMNGNVRELENGY

QQNDRNYPSQMPFTFRVHPIQPNLQEQ

>CL10716.Contig2

MVRGKTVLQRIENPTSRQVTFSKRRNGLLKKAFELSVLCEAEVGLIVFSPSGKLYEFAST

SMQETIDRFNGYNKGLLTNNKVKENTQLLKNPAVSMTRKIETLEAYRRKLLGEDLQSCST

DELQDMENQLEKSLSLIREKKNELYREKIQHLKEKEKILSEEIAFLREKSESCSWNPSTP

KHLQLLPCSQNSETSVVENEVTPESHPSYDSEVETELFIGRPDSRRVGDSCGKGL

>CL20429.Contig3

MGRGKVEMKRIENNINRQVTFAKRRNGLLKKAYELSVLCDAEIALIIFNSRGKLYEFCSS

SSILKTLEKYQRSTSSPSQSTTPPKNPEDETYQDYLKLKSRYEVLQQNQRNLNGLDLEPL

SVKELDQLENQLEGSLREIRSTKTQFMMDHLTELKTKEKELKEINKAMNRKVSTVTTFQQ

LEESKKMMIPQLKWEPEEHKVFYNHHGSASVDATSEGFYHPIQCNSTMHIGNSSSMRTNV

STGPTPQQSAIEYFPGWMI

>CL10616.Contig2

MGRGKIEIKRIENATNRQVTFSKRRSGIFKKARELSVLCDAEVSLIIVSSTNKVSDYISP

STTAKKIFDKYQEVEKIDLWVSHYEELQENLNHQKEINSKLRKEIRQRKGEDLNELSIKE

LRGLEQNLATSLETVRNRKFHVLSTQTDTCRKKIRSLEDAYKTNLRIAEERDEESHYVGP

NLRIAEERDEESHYVGPDNEDDDHYASSSITRLSNYGGGGSYLSFHLKPRQPSLQNAAGE

GYSYNGIPLLE

>CL7220.Contig1

MGRGKIEIKRIENSTNRQVTYSKRRNGIIKKAKEITVLCDAKVSLVIFSSTGKMSEYCSS

TNTGESRLIEILDRYQKSSGKKLWDAKHEYLSAEVDRIKKENDSMQIELRHLKGEDITSL

QPKELIPIEKALQDGFDSVKAKVSEIWKMHKRNLRLAEEEQNRLIFKCQQQQIEIDEVVR

DQMAVNGTGGGGYDHKAAAGTDYPPSHHHQRQMPFAFRGQPLQPNLHNHNK

>Unigene32254

MGRGRVQLKRIENKINRQVTFSKRRSGLLKKAHEISVLCEADVALIVFSVKGKLFEYSTN

DSMERILERYERYSFASREPVATDPDSQGNISMEYNKLKSKIEAMQKSQRHFMGEDIDAL

SFKELQNLEQQLDSALRQIRSRKGKALEEENNKLRKEVKEKELTLSICEQHHVENSPPIP

PLEALPDMKTSGEEDGDIVIVEAQPEPEPEPIHKTLMPPWMLRHIQGI

>CL18186.Contig4

MGRGKIEIKRIENTTNRQVTFCKRRNGLLKKAYELSVLCDAEVALIVFSSRGRLYEYSNN

SVKKTIERYKKASTDTSLPGSVSELNAQHYQQEAKKLRDTIGNLQNHNRHMLGEGISALN

LRELKALEKKIELGISKVRSKKEIDLQNDNMYLRAKIAENERTQHHMSLMPATEYEVISS

APFDSRNFLQVNLLEPNNNYSRSDQTTLQLG

>CL28920.Contig1

MVREKIQIKKIDNTTARQVTFSKRRRGLFKKAHELSILCDAEVAVVIFSATGKLFEYSSS

SMNEILERHKLHSKNLEKLERPSLELQLENSNYARLSKEISDKSHQLRQMRGEELRGLNI

EELQQLEQSLETGLSRVLETKSDKIMKEINTLHSKGIQLMEENERLRQMVDLSKTQTQFD

GESGIVVIEEGGQSSESVTNISNSGGQPPDNDSSDTSLKLGLSI

>Unigene7086

MGRGRVELKRIENKINRQVTFSKRRNGLLKKAYELSVLCDAEVGLIIFSSRGKLYEFASS

GMSRTLERYQRSSYNSHDNALAVVDRETQSWYQEVSKLKLKYEALQRSHRHLLGEDLGPL

NVKELQNLEKQLEGALTQARHRKTQIMMEQMEELRRKERQLGDMNKQLKHKYQLETDAHG

FRAQWESTALAANNFSMHPSQTSSMDCDATLQIGYHQYDGGDIPRSAGENNFIQGWPL

>CL28019.Contig1

MGRGRVELKRIENKINRQVTFAKRRNGLLKKAYELSVLCDAEVALIIFSNRGKLYEFCSS

SSMLKTLERYQKCSYGGPEPNVSAREAQEHSSHQEYLRLKARVEALQRSQRNLLGEDLAP

LSGKELEALERQLDSSLKQIRSTRTQYMLDQLTDLQRKEQLLSEANKTLRRRLEEGSQPN

PQQWDHNMQNVGYGRQHAQAQGEGFFHPLECAEPTLQIGYHNEQINVATAGPSMNNYMHA

QGWLA

>Unigene1341

MGRGKIEIKRIENATNRQVTYSKRRAGILKKAAELTVLCDAEVSLIMISNTGKLTEFFSP

NITPKGFFDKYQHLTGVDLWQSHYDRLQDNLMKLKEINAKLRREIGQRVGEDLSGLSLNE

LCGLEQHLQSSVKIVSKRKYKLIATQTDTYKKKVRNLQEINTNLVHEFEERFEDSYDIVN

HEAMSALELANVGAHIYAYRLQPSQPNLHDDGAYGLHDLRLG

>CL3032.Contig1

MGRGRVQLKRIENKINRQVTFSKRRSGLLKKAHEISVLCDAEVALIVFSTKGKLFEYSTD

SGMDKILERYERYSFAERDLVATDTDSQGNWSLEYTKLKSKIEILQKNQRHFMGENLDNM

SLKELQNLEQQLDTALKQIRSRKNQLMCESISDLQRKEKALQEQNNQLGKQLKEKEKQLA

QQGQWEQANHDQISSSQSLVLGQSIPSLNNGGNYQPRSTGSEEEVPRAQARPSSLMPAWM

LRHLNE

>CL3032.Contig3

MGRGRVQLKRIENKINRQVTFSKRRSGLLKKAHEISVLCDAEVALIVFSTKGKLFEYSTD

SGMDKILERYERYSFAERDLVATDTDSQGNWSLEYTKLKSKIEILQKNQRHFMGENLDKM

SLKELQNLEQQLDTALKQIRSRKNQLMCESISDLQRKEKALQEQNNQLGKQLKEKEKQLA

QQGQWEQANHDQISSSQSLVLGQSIPSLNNGGNYQPRSTGSEEEVPRAQARPSSLMPAWM

LRHLNE

>Unigene123412

MGRGKIEIKRIENATNRQVTYSKRRAGIMKKARELTVLCDAEVSLIMFSSTGKFSEYISP

STSPNPTKRIFDRYQQVSGINLWNSHYESMQNHLNKLKEVNNRLRKEIRQRMGEDLDELN

IDELRSLEQNLDTSVKVVRDRKNHVITTQTDTYKKKIRSLTESHHNLLREFEAGRDDESH

YALANQEDEYEAALELANGGPNIFAFRLQPSQPNLHDGRGYGSHDLRLA

>CL18186.Contig5

MGRGKIEIKRIENTTNRQVTFCKRRNGLLKKAYELSVLCDAEVALIVFSSRGRLYEYSNN

SVKKTIERYKKASTDTSLPGSVSELNAQHYQQEAKKLRDTIGNLQNHNRHMLGEGISALN

LRELKSLEKKIELGISKVRSKKNELLFNEIEYMQKREIDLQNDNMYLRAKIAENERTQHH

MSLMPATEYEVISSAPFDSRNFLQVNLLEPNNNYSRSDQTTLQLG

>CL18186.Contig3

MGRGKIEIKRIENTTNRQVTFCKRRNGLLKKAYELSVLCDAEVALIVFSSRGRLYEYSNN

SVKKTIERYKKASTDTSLPGSVSELNAQHYQQEAKKLRDTIGNLQNHNRHMLGEGISALN

LRELKSLEKKIELGISKVRSKKNELLFNEIEYMQKREIDLQNDNMYLRAKIAENERTQHH

MSLMPATEYEVISSAPFDSRNFLQVNLLEPNNNYSRSDQTTLQLG

>CL18186.Contig6

MGRGKIEIKRIENTTNRQVTFCKRRNGLLKKAYELSVLCDAEVALIVFSSRGRLYEYSNN

SVKKTIERYKKASTDTSLPGSVSELNAQHYQQEAKKLRDTIGNLQNHNRHMLGEGISALN

LRELKSLEKKIELGISKVRSKKNELLFNEIEYMQKREIDLQNDNMYLRAKIAENERTQHH

MSLMPATEYEVISSAPFDSRNFLQVNLLEPNNNYSRSDQTTLQLG

>Unigene119442

MGRGRVELKRIENKINRQVTFAKRRNGLLKKAYELSVLCDAEVALIIFSNRGKLYEFCSS

SSMFKTLERYQKCNYGQPEANVSARESLEHSSHQEYLKLKARVEALQRSQRNLLGEDLGP

LSGKELESLERQLDMSLKQIRSTRTQYMLDQLTDLQRREQMLSEANKNLRRRLEEGNQAS

QQHQWEQQMHAAGYGRQPAHATAEGFFHPIECEPTLQIGFQQDQITVAASGPSMSSYMPG

WLA

>CL10680.Contig1

MGRVKLKIKRLENTSGRQVTYSKRRAGILKKAKELSILCDIDIALLMFSPTGKPTLCLGE

RSTIEEVIAKFAQLTPQERAKRKLESLEALKKTFKKLDHDVNIQEFLGSSTQTVEDLTSQ

SRLLEGQLSEMHKRLSCWTEPEKINNLEHLRAMEESLRESLHRIRTHKENLGKQQLLPLD

CTGQFQNAMHLPLGMGGEQQVQPISWIPSSDGQHLMLPEDPNLLHQRDMECPTEASVPGY

SGYFGPPKQVEINPSGQNGGYFDPSKQVEIDTSGQNGGGGGYFGPTKQLEIDTCGHELNR

NACLRVQLGGQYPYPQQYGLNFLLDNKNMKSPEVKMNIQQSQSQSQSPLDYQVVANGGYD

INRPTWGPNTVVPCANGLFDDHMYPQQHH

>CL18653.Contig2

MAREKIQIKKIDNTTARQVTFSKRRRGLFKKAEELSVLCDAEVALIIFSATGKLFEYSSS

SMKEILERHNLHSKNLQKLAQPSLELQLENSSYARLTKEVAEKSHRLRQMRGEELRGLNI

EDLQQLEKSLEIGLGRVLETKGERIMKEIDTLQRKEMQLMEENERLRHQNVVQEEGQSSD

SVTNVVSNDNNNSSAGPPPREYDSSDTSLKLGLPCSS

>Unigene30723

MGRGKVELKRIENKINRQVTFAKRRNGLLKKAYELSVLCEAEVALIVFSTRGKLYEFSSC

SSILKTLERYQKCSYAAAESSTSTQETPHDYHEYLRLKQRVELLQQTQRNLLGEELGSLD

INGLEQLEHQLETSLKQVRSTKNHFMLDQLSDLQKKGEDLHEANRSLKKKLDECSAENAL

RLSWDANQQSIQYDCQAAPSEEFFQPLECNSTWHIRYNNGDQGQMPVATTAQNVHGFFPG

WML

>CL27078.Contig1

MGRGKIEIKRIENVTNRQVTYSKRRTGLIKKAMELAVLCDAEVSLMMFSSTGKLSEYISP

NTTQKRMFDKYQRVSGAELWNIHYEKMQVSLKQQKEVNMKLRKEIRQRMGEGLDDMNFEE

LRSLEQDLDASAKVVRDRKYHLIATQTETHRKKLRNLQETHTHLVREMEARGEDPYYEGD

YETYIGGANGGTHIITYRLQPSQAKGHEGAEDYGIYNLRLA

>CL21067.Contig2

MARGKVQMKRIENLVHRQVTFCKRRAGLLKKAKELSILCDADVGVIIFSDHGKLYELSTK

GSMQGLLDKYMKSSKGAQVSDEEANQPIMESEEEIALLKQEIQLLQKGLRYAFGGGSSGT

MSLDELELLEKHLELWIHNIRSTKREIMFQEIQLLKNKEGILKAANEYLQGKMEEQYYDI

PLNLTGIPYQLTIPDDIF

>Unigene119430

MGRGRVQLKRIENKINRQVTFSKRRSGLLKKAHEISVLCDAEVALIVFSTKGKLFEYSTD

SGMERILERYERYSYAERELVASELDQGNWSLEYSKLKAKIEILQKNQRHFMGEDLESMS

IKELQNLEQQLDTALKQIRSRKNQLMYESISELQKKEKVLQEQNNQLGKKLKEKEKEVAQ

QQAQWDQQNQGQSSPSFLLSQSLPSLNIGGSYHVRGRNGSEEEVVRTQTTRTNTSLMPPW

MIRHVNE

>CL28194.Contig1

MAREKIQIKKIDNTTARQVTFSKRRRGLFKKAGELSILCDADVALIIFSATGKLFEYSSS

SMKEILERHNLQSKNLQNLDQPSLELQLENGNCARLSKEVVERSRHLRNMRGEELQGLNI

EELQQLEKSLETGLGCVLETKSNRIMNEISTLQAKEKQLMEENERLRQQMMEMSKAPKNV

AAIESDNVVHEEGQSSESVTNICSSGGPPQDYDSSDTSLKLGLPFSS

>CL20429.Contig2

MGRGKVEMKRIENNINRQVTFAKRRNGLLKKAYELSVLCDAEIALIIFNSRGKLYEFCSS

SSILKTLEKYQRSTSSPSQSTTPPKNPEDETYQDYLKLKSRYEVLQQNQRNLNGLDLEPL

SVKELDQLENQLEGSLREIRSTKTQFMMDHLTELKTKQLEESKKMMIPQLKWEPEEHKVF

YNHHGSASVDATSEGFYHPIQCNSTMHIGNSSSMRTNVSTGPTPQQSAIEYFPGWMI

>CL2264.Contig2

MGRGKIEIKKIENTTNRQVTFSKRRAGLIKKARELSVLCDAQLGLIIFSSSGKLFEYHSE

HHSMREIIDRYQKVSGMQVREYNNQEIHTEVMRMREQNDRLQAGMRQYTGDNLTALDLDE

LNQLEQQLEIAVNKVRLRKDQLLKQQLDNLRRKEQLLEEQNSHYCRCIMEQQAAVEQHNA

MLEHKHVDQPFLEHLGVYSDEQARNLLQLSPLSPQLQTFRLQPTQPNLQEASLAYPRLQL

-

>Unigene11162

MGRGRVQLKRIENKINRQVTFSKRRSGLLKKAHEISVLCEADVALIVFSVKGKLFEYSTN

DSMERILERYERYSFASREPVATDPDSQGNISMEYNKLKSKIEAMQKSQRHFMGEDIDAL

SFKELQNLEQQLDSALRQIRSRKNQLTCVSITELQRKGKALEEENNKLRKEVKEKELTLS

ICEQHHVENSPPIPPLETLPDMKTSGEEDGDIVIVEAQPEPEPEPIHKTLMPPWMLRHIQ

GI

>CL19409.Contig1

MGRVKLKIKRLENTSGRQVTYSKRRAGILKKARELSILCDIDIALLMFSPTGKPTLCLGE

RSNIEEVIAKFAQLTPQERAKRKLESLEALKKTFKKLDHDVNIQDFLGTSTQTVEDLTNQ

SRLLQAQLSEMHKRLSYWNDLEKINNVDHLRAMEESIKESLNRIRTHKENLGKQQLMTLE

CAQFQNGMHLPLGMGGEQQPQTLSWISSNDSQHVMLSEDTNLLPQRDMECSTDPSLHSYS

GYLGTGKQVEIDNSGQDSGSLHELSRDACLRLQLGAQFPYPPYNLNLMNDKKYKADGKMS

LQEPPMEYQVSSSSFEPPRHRYDASNSGWASTSGHCAVAMFDGSSYPHAQQPN

>CL3032.Contig2

MGRGRVQLKRIENKINRQVTFSKRRSGLLKKAHEISVLCDAEVALIVFSTKGKLFEYSTD

SGMDKILERYERYSFAERDLVATDTDSQGNWSLEYTKLKSKIEILQKNQRHFMGENLDNM

SLKELQNLEQQLDTALKQIRSRKNQLMCESISDLQRKEKALQEQNNQLGKQLKEKEKQLA

QQGQWEQANHDQISSSQSLVLGQSIPSLNNGGNYQPRSTGSEEEVPRAQARPSSLMPAWM

LRHLNE

>CL10523.Contig3

MAREKIQIRKIDNATARQVTFSKRRRGLFKKAEELSILCDADVALIIFSATGKLFDYSSS

NMKEILERHNLHSKNIQKLEQPSLELQLENSNCARLQKEVTEQSHRLRQMRGEELQALNI

EELQQLEKVLEAGLSRVLETKGERIMNEINTLQSKGKELLEENERLRQQMTDITKGQSQR

PVVINCENVVHEEGQSSESVTNVCGSMGPPQDDDSSDTSLKLGLPFSS

>Unigene152236

MGRGKIEIKRIENTTNRQVTFCKRRNGLLKKAYELSVLCDAEVALIVFSSRGRLYEYSNS

SSIKSTIDRYKKACADSSNTTSIVEANAHYYQQEATKLKQQIQILQNSNRHLMGDSLSSL

TVKELKQLENRLERGLTRIRSKKHEMLFAEIEYMQRREVELQKENMFLRAKVAENEHAEQ

QQTSMVPASDFEPIQTYDSRNYFHVNMLDGGPSVSYSHPDQTALHLG

>CL18653.Contig1

MAREKIQIKKIDNTTARQVTFSKRRRGLFKKAEELSVLCDAEVALIIFSATGKLFEYSSS

SMKEILERHNLHSKNLQKLAQPSLELQLENSSYARLTKEVAEKSHRLRQMRGEELRGLNI

EDLQQLEKSLEIGLGRVLETKGERIMKEIDTLQRKEMQLMEENERLRHQMLGISKSRKCA

VVDHSSQNVVQEEGQSSDSVTNVVSNDNNNSSAGPPPREYDSSDTSLKLGLPCSS

>Unigene158374

MGRGRVELKRIENKINRQVTFAKRRNGLLKKAYELSVLCDAEVALIIFSTRGKLYEFCST

SSMLTTLERYQKCSYSALEASTSNKELQSSYQEYLKLKSRVELLQRTQRNLLGEELGSLS

TKELEQLEQQLDTSLRQIRSTKTQFMLDQLSDLHKKEQLLQDANKALKRKLEETNATNSL

RLSWETGGQNIHYNRQPPQSEGFFQPLECNSSLQIGYNSVGTDQINIANSSQNVSGFIPG

WML

>CL10523.Contig1

MAREKIQIRKIDNATARQVTFSKRRRGLFKKAEELSILCDADVALIIFSATGKLFDYSSS

NMKEILERHNLHAKNLQKLEQPSLELQLENSNCGRLQKEVTEQSHRLRQMRGEELQALNI

EELQQLEKVLEAGLSRVLETKGERIMNEINTLQSKGKELLEENERLKQQMSDITKGQRPV

VINCENVVHEEGQSSESVTNVCGSMGPPQDDDSSDTSLKLGLPFSS

>Unigene146375

MARGKVQLKRIENPVHRQVTFCKRRSGLLKKVKELSVLCDSDIGIIIFSTHGKLYELSTK

GTMQYMIERYKETSQGAQIAEGEPKQSLESEKEISMMKQEIQHLQKGLRYMFGGGTGIVK

LDELDALEKHLELWISNIRLTKMQIMFQEIQMLKNKIEEQTEIFDISLLMTEFPYTK

>Unigene114330

MGRGRVQLKRIENKINRQVTFSKRRTGLLKKAHEISVLCDADVALIVFSTKGKLFEFSSD

TSMDSILERYERQCFAEEELKASHPESEGNWSLEHTKLKARIETLQKNQRHFMGEDLDPM

SLKELQNLEHQLDTALKHIRSRKNQLLYASIAELRKKEKALHEQNNLLGKKIKEKEELIA

KWPVQQNEAENPPSFLTQALPSLTLRTGTYQTGKVVGGEEERARPNMIMPPWMLGHVNQ

>CL10523.Contig2

MAREKIQIRKIDNATARQVTFSKRRRGLFKKAEELSILCDADVALIIFSATGKLFDYSSS

NMKEILERHNLHAKNLQKLEQPSLELQLENSNCGRLQKEVTEQSHRLRQMRGEELQALNI

EELQQLEKVLEAGLSRVLETKGERIMNEINTLQSKGKELLEENERLKQQMSDITKGQRPV

VINCENVVHEEGQSSESVTNVCGSMGPPQDDDSSDTSLKLGLPFSS

>CL19409.Contig6

MGRVKLKIKRLENTSGRQVTYSKRRAGILKKARELSILCDIDIALLMFSPTGKPTLCLGE

RSNIEEVIAKFAQLTPQERAKRKLESLEALKKTFKKLDHDVNIQDFLGTSTQTVEDLTNQ

SRLLQAQLSEMHKRLSYWNDLEKINNVDHLRAMEESIKESLNRIRTHKENLGKQQLMTLE

CAQFQNGMHLPLGMGGEQQPQTLSWISSNDSQHVMLSEDTNLLPQRDMECSTDPSLHSYS

GYLGTGKQVEIDNSGQDSGSLHELSRDACLRLQLGAQFPYPPYNLNLMNDKKYKADGKMS

LQEPPMEYQVSSSSFEPPRHRYDASNSGWASTSGHCAVAMFDGSSYPHAQQPN

>CL28920.Contig2

MVREKIQIKKIDNTTARQVTFSKRRRGLFKKAHELSILCDAEVAVVIFSATGKLFEYSSS

SMNEILERHKLHSKNLEKLERPSLELQLENSNYARLSKEISDKSHQLRQMRGEELRGLNI

EELQQLEQSLETGLSRVLETKSDKIMKEINTLHSKGIQLMEENERLRQMVDLSKTQTQFD

GESGIVVIEEGGQSSESVTNISNSGGHPPDNDSSDTSLKLGLSI

>CL2728.Contig3

MGKRKIDIVRIEKPETRHVTFSKRRKGLFGKAAKFSNDFGIDVSLIVFSPGGKPFAFGPI

ETVVERILGNGCEGSNNEEEQMRQWWLEFMDIEKQESKCYDLESLWYVRGKVETLRDKVR

ALLNDASGSSSSSSFNAFTSDNNTVAVAPLSPRADSATAIIDFDDGYGQRLEDYSTSTSN

VVDCWGVALKGKSKEDDGPLLLPYVDIDSEFDVSEFFNFNTEGEKS

>CL2728.Contig4

MGKRKIDIVRIEKPETRHVTFSKRRKGLFGKAAKFSNDFGIDVSLIVFSPGGKPFAFGPI

ETVVERILGNGCEGSNNEEEQMRQWWLEFMDIEKQESKCYDLESLWYVRGKVETLRDKVR

ALLNDASGSSSSSSFNAFTSDNNTVAVAPLSPRADSATAIIDFDDGYGQRLEDYSTSTSN

VVDCWGVALKGKSKEDDGPLLLPYVDIDSEFDVSEFFNFNTEGEKS

>Unigene137450

MVRGKTQMKRIENATSRQVTFSKRRNGLLKKAFELSVLCDADVALIVFSPRGKLYEFSSS

SIQTTIERYQKYTKDIEVENKEVDQQNTQHLKYEAANLAKKIEMLDGSKRKLMGEGLESC

SIDDLQQVENQLERSLSHIRARKSQLFREKIEQLKEKEKALLEENKMLTEKVCIFLLLT

>CL2728.Contig2

MGKRKIDIVRIEKPETRHVTFSKRRKGLFGKAAKFSNDFGIDVSLIVFSPGGKPFAFGPI

ETVVERILGNGCEGSNNEEVQMRHWWLEFMDIEKQESKCYDLESLWYVRGKVETLRDKVR

ALLNDASGSSSSSSFNAFTSDNNTVAVAPLSPRADSATAIIDFDDGYGQRLEDYSTSISN

AVDCWGVALKGKSKEDDGPLLLPYVDIDSEFDVSEFFNFNTEGEKS

>CL27155.Contig5

MGRGKVEMKRIENKINRQVTFAKRRNGLLKKAYELSVMCDAEVALIMFSSRGKLYEFCSS

SSMRKTLERYQQCSYTALEASKLAKDTNQASYQEYLSLKEKFEFLRQSQRNLHGEDLESL

SMKELGQLEQQLESSLKHVKSTLNDIMLDQLSELQMMEQMLREANKSLQRKLEESTSKDP

LQLL

>CL27155.Contig4

MGRGKVEMKRIENKINRQVTFAKRRNGLLKKAYELSVMCDAEVALIMFSSRGKLYEFCSS

SSMRKTLERYQQCSYTALEASKLAKDTDQASYQEYLSLKEKFEFLRQSQRNLHGEDLESL

SMKELGQLEQQLESSLKHVKSTLNDIMLDQLSELQMMEQMLREANKSLQRKLEESTSKDP

LQLL

>CL27155.Contig2

MGRGKVEMKRIENKINRQVTFAKRRNGLLKKAYELSVMCDAEVALIMFSSRGKLYEFCSS

SSMRKTLERYQQCSYTALEASKLAKDTDQASYQEYLSLKEKFEFLRQSQRNLHGEDLESL

SMKELGQLEQQLESSLKHVKSTLNDIMLDQLSELQMMEQMLREANKSLQRKLEESTSKDP

LQLL

>CL27155.Contig1

MGRGKVEMKRIENKINRQVTFAKRRNGLLKKAYELSVMCDAEVALIMFSSRGKLYEFCSS

SSMRKTLERYQQCSYTALEASKLAKDTDQASYQEYLSLKEKFEFLRQSQRNLHGEDLQSL

SMKELGQLEQQLESSLKHVKSTLNDIMLDQLSELQMMEQMLREANKSLQRKLEESTSKDP

LQLL

>CL2728.Contig1

MGKRKIDIVRIEKPETRHVTFSKRRKGLFGKAAKFSNDFGIDVSLIVFSPGGKPFAFGPI

ETVVERILGNGCEGSNNEEEQMRQWWLEFMDIEKQESKCYDLESLWYVRGKVETLRDKVR

ALLNDASGSSSSSSFNAFTSDNNTVAVAPLSPRADSATAIIDFDDGYGQRLEDYSTSISN

AVDCWGVALKGKSKEDDGPLLLPYVDIDSEFDVSEFFNFNTEGEKS

>CL3350.Contig5

MGRGRVQLKRIENKINRQVTFSKRRSGLLKKAHEISVLCEADVALIVFSVKGKLFEYSTN

DSMERILERYERYSFASREPVATDPDSQGNISMEYNKLKSKIEAMQKSQRHFMGEDIDAL

SFKELQNLEQQLDSALRQIRSRKGKALEEENNKLRKEVNFNIYSFGVLFTSQLSKNKHLL

KFMK

>Unigene74157

MGRGRVQLKRIENKINRQVTFSKRRSGLLKKAHEISVLCEADVALIVFSVKGKLFEYSTN

DSMERILERYERYSFASRELVATDPDSQGNISMEYNKLKSKIEAMQKSQRHFMGEDIDAL

SFKELQNLEQQLDSALRQIRSRKNQLTCVSITELQRKVDISHTFILVIEVLNKFKRL

>CL16956.Contig3

MGRVKLQIKRIENNTNRQVTFSKRRNGLIKKAYELSVLCDIDIALIMFSPSGRLSHFSGK

RRIEDVLTRYINLPDHDRGGSIQNREYLIRTLKKLKTESDMAIQIANPTAVNTHVEQLQQ

EITSFQQQIQITDEQLRNFEPDLLKLTSMDELDSCEKQLVEVLTRVSQRKNYLLSNHLSS

YDPSNLQMYLDSQVGMVPSTENKDVNWYLENGHNPNQIFVGPNSLIPLRDQQLAMYDPMS

QGTSLNVDPRSIEACQISNQNDQAVPLWHQVYTSTELLNALVPPGAFPLFQNEMEGPDIS

SMMPPEQVETLPNCTTVSTQNNDEVTYQSSLQQQIDVE

>CL26633.Contig2

MGRGRVQLKRIENKINRQVTFSKRRSGLLKKAHEISVLCEADVALIVFSVKGKLFEYSTN

DRYDVLGYLLYYSFKYRLLSSVCCFRT

>Unigene89536

MGRRKIAIEKIEDKTKLQITFTKRRNGLFKKAGELCFRCKSSQIAIIAFSPGGKIYSFGH

PNAELVIRRYVAERKRRLAATINRRDWKKVLRREWDDENIEAETDVQQLQQMFDSMMNLQ

NKITERLQEKDNSK

>CL27078.Contig2

MGRGKIEIKRIENVTNRQVTYSKRRTGLIKKAMELAVLCDAEVSLMMFSSTGKLSEYISP

NTTQKRMFDKYQRVSGAELWNIHYEKMQVSLKQQKEVNMKLRKEIRQRMGEGLDDMNFEE

LRSLEQDLDASAKVVRDRKYHLIATQTETHRKKVSVFNHFYLEKT

>CL13903.Contig3

MGRGRVELKRIENTTNRQVTFSKRRNGLIKKAFELSVLCDAEVALVIFSPTGKVFEFSSH

DMNRTIARYRNKSGLPQLCDELSRSVEENWYDIYMGET

>CL7507.Contig1

MGRGKIEIKRIENLTNRQVTYSKRRNGILKKAKEITVLCEAQVSLVIFSSTGKMAEFSSS

PMITILDKYQTSSGNKLWDAKH

>CL13903.C1

MGRGRVELKRIENTTNRQVTFSKRRNGLIKKAFELSVLWDAEVALVIFSPTGKVFEFSSH

DMNRTIARYRNKSGLPQLCDELSRSVEVGIRFKMIILLVRGHP

>CL13903.C2

MGRGRVELKRIENTTNRQVTFSKRRNGLIKKAFELSVLWDAEVALVIFSPTGKVFEFSSH

DMNRTIARYRNKSGLPQLCDELSRSVEVNIYAWYNAISEKIYSSFILLRC
