# Supplementary figures and images for: Identification and Characterization of MIKCc-Type MADS-Box Genes in the Flower Organs of Adonis amurensis
Source: Int J Mol Sci. 2021 Aug 28;22(17):9362. doi: 10.3390/ijms22179362 (PMC8430553; doi:10.3390/ijms22179362)

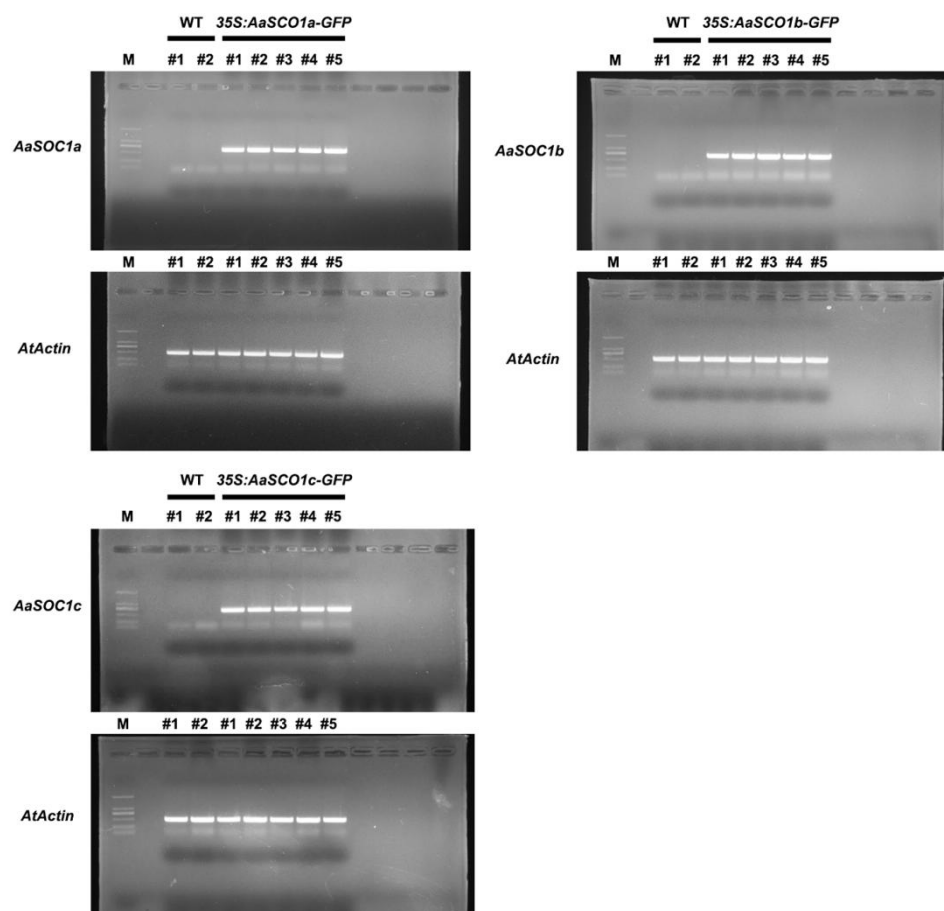

Figure S1. PCR original detection image.

Supplement: Supplementary file 1 [file ijms-22-09362-s001.zip › Supplementary Figure S1.pdf]
